# An unusual case of primary splenic soft part alveolar sarcoma: case report and review of the literature with emphasis on the spectrum of *TFE3*-associated neoplasms

**DOI:** 10.1186/s13000-024-01483-4

**Published:** 2024-04-20

**Authors:** René Guérin, Anne-Lise Menard, Emilie Angot, Nicolas Piton, Pierre Vera, Lilian Schwarz, Jean-Christophe Sabourin, Marick Laé, Pierre-Alain Thiébaut

**Affiliations:** 1 Department of Pathology, Rouen University Hospital, Rouen, France; 2 Department of Hematology, Centre Henri Becquerel, Rouen, France; 3 Department of Nuclear Medecine, Centre Henri Becquerel, Rouen, France; 4 Department of Digestive Surgery, Rouen University Hospital, Rouen, France; 5 Department of Pathology, Centre Henri Becquerel, Rouen, France

**Keywords:** Alveolar soft part sarcoma, Splenic, TFE3

## Abstract

**Background:**

Alveolar soft part sarcoma is a rare tumour of soft tissues, mostly localized in muscles or deep soft tissues of the extremities. In rare occasions, this tumour develops in deep tissues of the abdomen or pelvis.

**Case presentation:**

In this case report, we described the case of a 46 year old man who developed a primary splenic alveolar soft part sarcoma. The tumour displayed typical morphological alveolar aspect, as well as immunohistochemical profile notably TFE3 nuclear staining. Detection of ASPSCR1 Exon 7::TFE3 Exon 6 fusion transcript in molecular biology and TFE3 rearrangement in FISH confirmed the diagnosis.

**Conclusion:**

We described the first case of primary splenic alveolar soft part sarcoma, which questions once again the cell of origin of this rare tumour.

## Background

Alveolar soft part sarcoma (ASPS) was initially described in 1952 by Christopherson WM [1] and is referred to as uncertain differentiation tumour in the 5th edition of the WHO classification of soft part tumours [2]. This tumour frequently arises in muscles or deep soft tissues of the extremities, notably in the lower limb (51%) [2]. Rare locations, such as pelvis, genital tract and bladder, were also described [2]. Metastatic evolution is frequent and often concomitantly discovered at diagnosis [3], essentially in the lungs, liver, bones and brain [2]. Exceptional cases of splenic metastases were also described in the literature [4, 5]. However, to our knowledge, no primary splenic ASPS have ever been reported. Here we report the case of a 46 year old patient who developed a primary splenic ASPS and we review the current knowledge of this rare tumour, emphasizing the discussion on the cell of origin.

## Case presentation

**Fig. 1:**
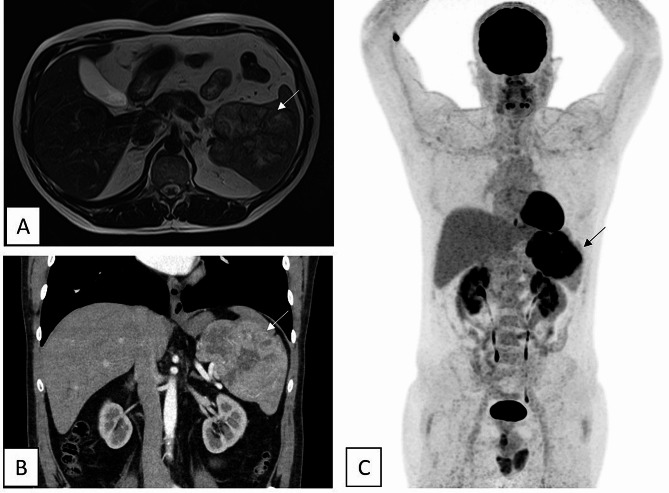
Imaging evaluation after 4 years of the initial symptomatology showing a 106 mm lobulated heterogenic and hypermetabolic splenic mass. **A** : Axial abdominal MRI. **B** : Frontal abdominal CT-scan. **C** : Whole body TEP-scan (SUVmax = 16.9). Arrows indicating splenic tumour mass with no other identified lesion

In this case report, we describe the case of a 46 year old man, with no significant personal, nor familial history, who consulted for persistent abdominal pain. Initial ultrasound and CT-scan showed no apparent lesion. During the follow up, an abdominal MRI (Fig. 1A) and CT-scan (Fig. 1B) were performed after 4 years of symptomatology and showed a 10 cm-heterogeneous splenic mass, which was strongly hypermetabolic on the PET-scan (SUVmax = 16.9) (Fig. 1C).

A diagnostic splenectomy was collegially decided due to the high probability of a neoplastic etiology.

**Fig. 2:**
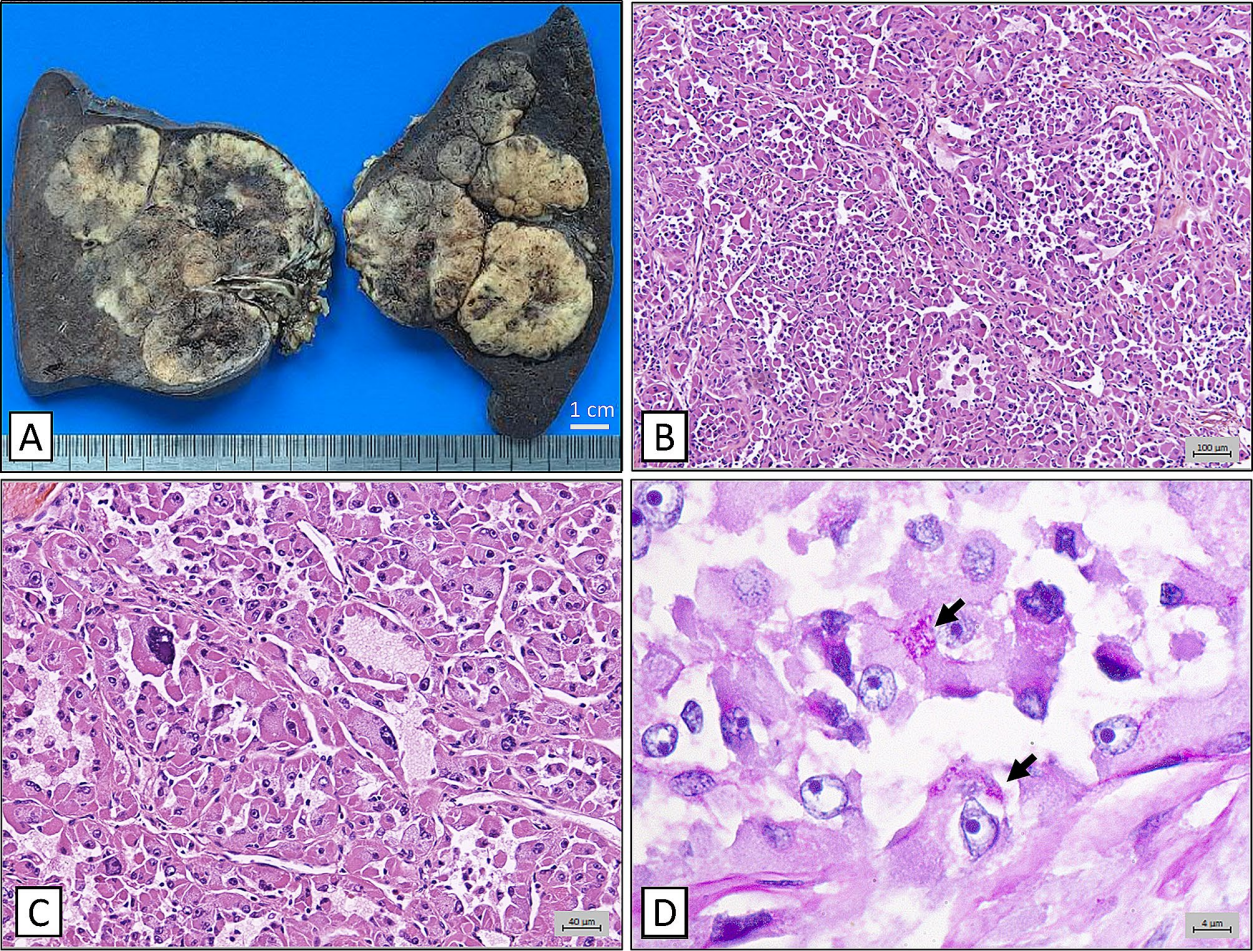
Pathology analysis of the splenic specimen. **A** : Macroscopic appearance of the splenic lesion (after formaldehyde fixation). Note the proximity of the tumor with the large diameter vessels of the spleen. **B** : low magnification (x100) emphasizing pseudo-alveolar architecture. **C** : High magnification (x200) focally revealed marked nuclear atypia. **D** : High magnification (x630) showing intracytoplasmic diastase-resistant rod-like structures on the PAS coloration (black arrows). Bar scales indicate respectively 100, 40 and 4 μm

The splenectomy specimen weighted 588 g and measured 15 × 11 × 9 cm. Dissection revealed a unique peri-hilar 12 cm tumour, which was multilobulated, well delimited and of yellowish appearance. Splenic capsule was preserved and the excision was complete (Fig. 2A).

Microscopically, the tumour presented a uniform pseudo-alveolar architecture, made of closely packed-nests of tumour cells and separated by fibrous and highly vascularised septa. The nest centers showed discohesive cells, explaining the pseudo-alveolar pattern. Tumour cells were large, with ovoid nuclei containing a vesicular chromatin and prominent nucleoli. Tumour cell cytoplasms were large, eosinophilic and granulous (Fig. 2B-C).

PAS-diastase coloration revealed agglomerated rod-like intracytoplasmic structures (Fig. 2D).

Rare signs of vascular invasion were observed but excision was complete.

**Fig. 3:**
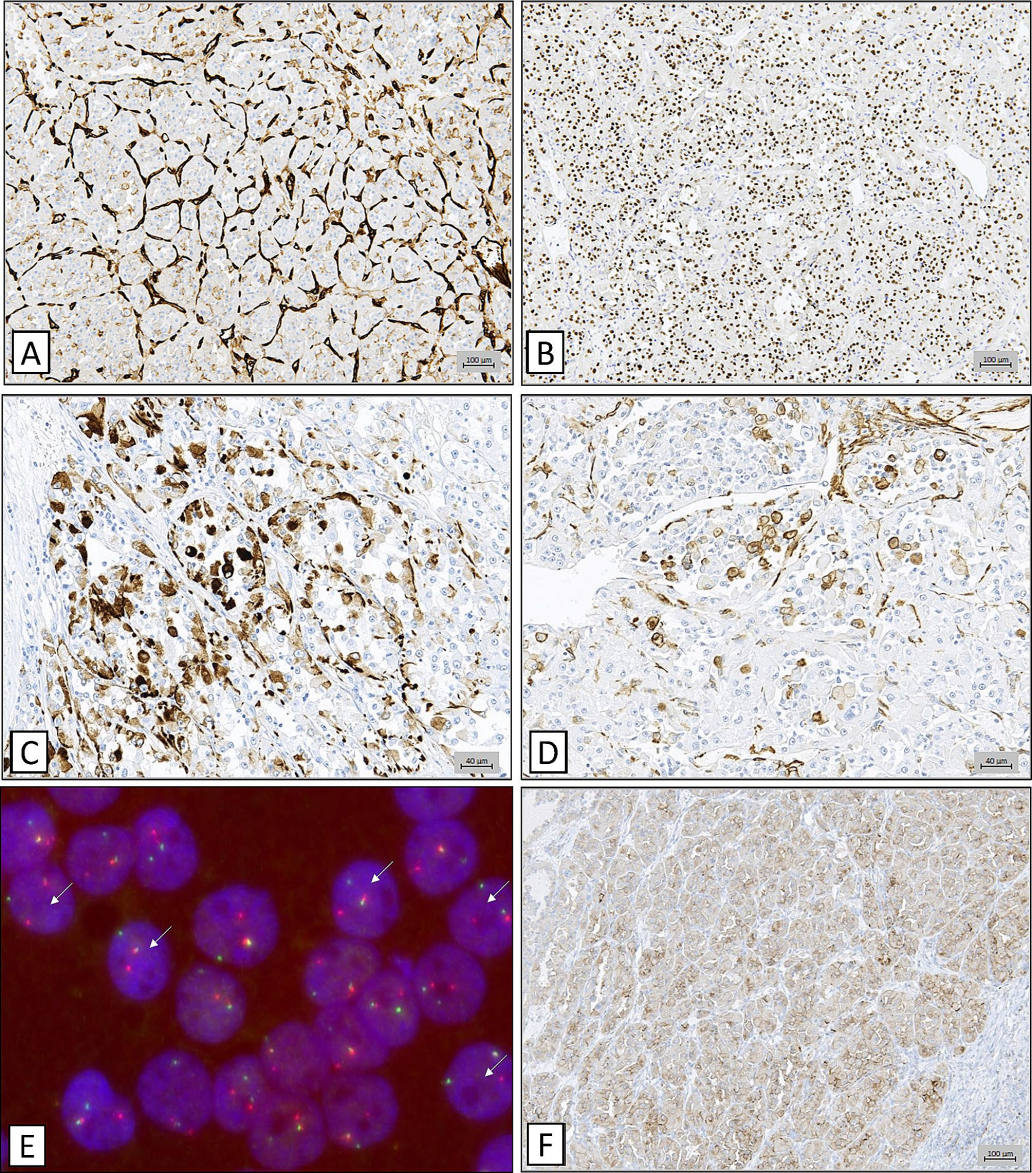
Immunohistochemical and FISH analysis of the splenic tumour. **A** : Highly vascularized pattern between tumour nests highlighted by CD31 immunostaining. **B** : Nuclear diffuse TFE3 immunostaining of tumour cells. **C-D** : Heterogenous staining with Smooth muscle actin and Desmin antibodies. **E** : Illustration of TFE3 rearrangement in FISH. White arrows indicating break apart (split) signal in the majority of nuclei. **F** : PDL1 staining showing a weak to moderate but diffuse staining of tumour cells (TPS score > 90% and CPS score at 95). Bar scales indicate 100 μm for **A**, **B** and **F**, 40 μm for **C-D**

Immunohistochemical analysis revealed a diffuse nuclear staining of tumour cells with TFE3 antibody (Fig. 3B). Of note, we observed a heterogeneous staining of tumour cells with Desmin and Smooth-muscle Actin (SMA) (Fig. 3C-D). Other muscle markers such as Caldesmon (smooth muscle), Myogenin (striated muscle) or MyoD1 (myoblastic marker) were all negative (not shown). Interestingly, vascular markers CD31 (Fig. 3A), CD34 and ERG highlighted a dense capillar network between tumour nests, without staining of tumour cells, which ruled out an endothelial origin such as angiosarcoma or littoral cell angioma. Other markers, such as PS100, MelanA, HMB45, SOX10, Chromogranin, Synaptophysin, CD56, PAX8 and a large panel of cytokeratins (CK7, CK20, panCK, MNF116), were all negative (not shown), eliminating melanoma, PEComa and carcinoma (notably renal cell carcinoma). Proliferation index reflected by Ki67 staining was low and estimated at 10% (not shown).

The combination of morphological analysis and immunohistochemical profile was suggestive of ASPS. However, due to the unusual splenic localization, a molecular confirmation by NGS-targeted LD-RT-PCR [6] was performed, allowing the detection of *ASPSCR1* Exon 7::*TFE3* Exon 6 fusion transcript. *TFE3* rearrangement was also confirmed by FISH, which showed a split signal in the majority of tumour cells (Fig. 3E).

Of note, anti-PDL1 staining showed a weak to moderate diffuse staining (TPS score > 90% and CPS at 95) (Fig. 3F).

Whole body extension check-up, comprising PET-CT and cerebral MRI, were negative and did not show any sign of extra-splenic secondary lesions.

## Discussion 

ASPS is a rare entity (less than 1% of soft part sarcomas [2]), essentially affecting young patients less than 30 years old and with a slight predominance in women [3]. This tumour usually develops in the deep tissues of limbs, with a slow and painless growth, which can evolve over the course of several years. Truncular lesions are rare (around 8%) and are associated with a worse prognosis [2]. To our knowledge, our case is the first described case of primary splenic ASPS, with no secondary lesion observed to this day.

With regards to the rarity of the lesion and location, we confirmed the diagnosis with several methods including NGS-targeted LD-RT-PCR, fluorescent in situ hybridization (FISH) and TFE3 immunohistochemistry (IHC), the latter showing a nucleus staining in more than 92% of cases [7], due to the rearrangement of *TFE3* (t(X;17)(p11;q25)) [8].

Differential diagnoses were all ruled out with extensive immunohistochemical analysis, including carcinomas (no cytokeratin or PAX8 staining), especially renal cell carcinoma which can display a similar morphology with large eosinophilic cytoplasm and atypical nucleolated nuclei and similar genetic alteration t(X;17)(p11;q25) [9]. TFE3 nuclear staining can also be observed in PEComa [10] and granular cell tumour [11] but MelanA, HMB45, PS100 and SOX10 are usually positive in these tumours, especially PEComa, which can sometimes share morphology closely resembling ASPS as well as alterations in the *TFE3* locus [10, 12–14]. Interestingly, recent studies described PEComa-like neoplasms that harbour the same *ASPSCR1-TFE3* translocation observed in ASPS [15]. These neoplasms however display a significantly different morphology than ASPS that was more reminiscent of PEComa with tight nests of tumour cells varying from epithelioid to spindle cell morphology in a hyalinized stroma. Importantly, these tumours lack the characteristic alveolar discohesive pattern and highly vascularized stroma observed in our case that highly suggested ASPS. Of note, in terms of immunohistochemical profile, these PEComa-like neoplasms showed no reactivity for MelanA or HMB45 but were positive for TFE3 and occasionally for SMA. Finally, using gene expression profiling and clustering, Wang et al. suggested the existence of a closely related entity originally thought to be derived from PEComa that was called melanotic XP11 neoplasm, which share morphological features with ASPS, except for strong melanin pigmentation [16]. Gene fusion partners of TFE3 were also different as well as immunohistochemical profile, which show positivity for MelanA and HMB45. Nevertheless, the authors showed that melanotic Xp11 neoplasms clusterized closer to ASPS than to PEComa, suggesting that melanotic Xp11 neoplasm could be a particular variant of ASPS, reinforcing the complexity to define these entities [16]. A final differential diagnosis could be with alveolar rhabdomyosarcoma, which can also display an alveolar pattern, but the diagnosis was ruled out by negativity of Myogenin and MyoD1 staining and no detection of *PAX*::*FOXO1* fusion by NGS [2].

ASPS cell of origin is controversial and was summarized in 2006 by Folpe et al [17]. ASPS was initially thought to be the malignant version of granular cell tumours (formerly referred to as granular cell myoblastoma), but the hypothesis of muscular origin was brought up by the ultrastructural similarity between the actin filaments observed in rhabdomyoma or nemalin myopathy and the intracytoplasmic crystals observed in ASPS. Of note, muscular markers are usually negative in ASPS, but some studies reported inconsistent focal positivity for muscle markers depending on the localization [18]. A recent report described a focal positivity for Desmin and SMA in two ASPS that unexpectedly harboured non-canonical translocation of *TFE3* with *HNRNPH3, DVL2* or *PRCC*, the latter two being previously described in renal cell carcinoma and PEComa [19]. This latest study strongly highlights a previously undescribed genetic diversity in ASPS and suggests once more that the same genetic alteration can lead to different neoplasms depending on the cell of origin in which the anomaly occurs.

In 2001, Ladanyi et al. detected the fusion transcript t(X;17)(p11;q25) [20] and showed immunohistochemical staining of intracytoplasmic crystals by MCT1 and CD147 antibodies [21]. Weiss et al. then suggested that MCT1, a monocarboxylate transporter protein, and CD147, a chaperone protein assisting MCT1 at cytoplasmic membrane, both presented an activation of their promoter by TFE3, thus leading to excessive protein deposit [22]. Unfortunately, to this day, the cell of origin remains uncertain and no benign or normal counterpart of this tumour has been discovered. Nevertheless, in the light of the recent studies developed earlier, Argani et al. suggested that these tumours could originate from a neural crest precursor that would differentiate toward mesenchymal or melanocytic cell, thus explaining features shared between PEComa and ASPS [15]. Interestingly, in our case, the hilar splenic development in a highly vascularized region and the heterogenous immunohistochemical positivity of muscular makers Desmin and SMA (Fig. 3C-D) could support a development from the vascular wall (smooth muscle cell, pericyte, etc.).

ASPS is classified as high grade (grade 3) according to the FNCLCC grading system [2]. Overall survival at 5 year is comprised between 60 and 100% for localized lesions and 20 to 46% for metastatic cases. Unfavourable prognostic factors include male, advanced age, size (above 5 cm if localized and > 10 cm if metastatic) and truncular location [23]. Treatment recommendations include complete surgical resection of the primary tumour and metastases (if possible), combined with radiation or chemo-therapy, which is often associated with mediocre benefits [8, 23].

Recently, evidence of potential response to checkpoint inhibitors was brought up, notably by a randomized clinical trial evaluating combination of anti-VEGF axitinib and anti-PD1 pembrolizumab. This trial pooled advanced sarcomas (including 11 ASPS) and showed partial response in 6 out of 11 patients and stability in 2 out of 11 patients (72.7% of clinical benefit) [24].

In our case, tumour cells showed weak to moderate but diffuse staining with anti-PDL1 antibody (TPS score > 90% and CPS at 95) (Fig. 3F), which could allow future anti-PD1 treatment in case of recurrence. Of note, no evident tertiary lymphoid structure was observed on the invasion front, as recommended by Italiano et al. as a predictive marker of immunotherapy response [25]. To this day, the patient is still considered in remission after surgical excision and no complementary treatment was initiated, notably no radiation therapy in this retroperitoneal location.

## Conclusion 

Herein we reported the first case of primary splenic ASPS, with typical morphology and confirmation by multiple methods (molecular biology, FISH and IHC). ASPS cell of origin remains uncertain but could be, in light of this case report, of vascular wall origin. The close resemblance with other TFE3-related tumours, such as PEComa, newly described PEComa-like neoplasms or other Xp11-associated neoplasms is intriguing and suggests similar oncogenic pathways arising in different cells of origin. Further investigation needs to be performed to understand the biology of this rare tumour.

## Data Availability

The datasets used and/or analysed during the current study are available from the corresponding author on reasonable request.

## Abbreviations

ASPS: Alveolar soft part sarcoma
FISH: Fluorescent in situ hybridization
IHC: Immunohistochemistry
SMA: Smooth muscle actin
